# Equitable resourcing of primary health care in remote communities in Australia’s Northern Territory: a pilot study

**DOI:** 10.1186/s12875-017-0646-9

**Published:** 2017-06-29

**Authors:** John Wakerman, Lisa Sparrow, Susan L Thomas, John S Humphreys, Mike Jones

**Affiliations:** 1 Flinders Northern Territory, Casuarina, Australia; 2 School of Rural Health, Monash University, Melbourne, Australia; 3 Psychology Department, Macquarie University, Sydney, Australia; 4 Centre for Research Excellence in Rural and Remote Primary Health Care, Flinders University, Bedford Park, South Australia 5042 Australia

**Keywords:** Primary health care, Remote, Equity, Access, Cost, Benchmark, Funding

## Abstract

**Background:**

Improved Primary Health Care (PHC) utilisation is central to reducing the unacceptable morbidity and mortality rates characterising populations living in remote communities. Despite poorer health, significant inequity characterises the funding of PHC services in Australia’s most remote areas. This pilot study sought to ascertain what funding is required to ensure equitable access to sustainable, high quality primary health care irrespective of geographical remoteness of communities.

**Methods:**

High performing remote Primary Health Care (PHC) services were selected using improvement measures from the Australian Primary Care Collaboratives Program and validated by health experts. Eleven PHC services provided data relating to the types of services provided, level of service utilisation, human resources, operating and capital expenses. A further four services that provide visiting PHC to remote communities provided information on the level and cost of these services. Demographic data for service catchment areas (including estimated resident population, age, Indigenous status, English spoken at home and workforce participation) were obtained from the Australian Bureau of Statistics 2011 census. Formal statistical inference (*p*-values) were derived in the linear regression via the nonparametric bootstrap.

**Results:**

A direct linear relationship was observed between the total cost of resident PHC services and population, while cost per capita decreased with increasing population. Services in smaller communities had a higher number of nursing staff per 1000 residents and provided more consultations per capita than those in larger communities. The number of days of visiting services received by a community each year also increased with population. A linear regression with bootstrapped statistical inference predicted a significant regression equation where the cost of resident services per annum is equal to $1,251,893.92 + ($1698.83 x population) and the cost of resident and visiting services is equal to $1,378,870.85 + ($2600.00 x population).

**Conclusions:**

The research findings provide empirical evidence based on real costs to guide funding for remote PHC services that takes into account the safety and equity requirements for a minimum viable service. This method can be used as a transparent, coordinated approach to ensure the equitable delivery of sustainable, high quality PHC in remote communities. This will in turn contribute to improved health outcomes.

## Background

Although Australia is ranked consistently in the top ten Organisation for Economic Co-operation and Development (OECD) countries for life expectancy, it has the third highest regional disparity. For example, the Northern Territory’s (NT) population of 244,900 people are spread over an area of 1.3 million square kilometres (more than twice that of France) and have a life expectancy 6.1 years less than those residing in the more densely populated Australian Capital Territory [1]. This disparity is largely due to the higher morbidity and mortality of populations living in remote communities, partly reflecting the high proportion of Aboriginal and/or Torres Strait Islander (hereafter as Indigenous) people resident there.

Reducing these unacceptable morbidity and mortality rates requires access to appropriate health services. The Primary Health Care (PHC) sector is crucial in preventing, detecting and managing illness and injury [2]. Improved PHC utilisation results in decreased morbidity and mortality, lower rates of hospitalisation and fewer costs [3]. Despite the high need for PHC in remote communities, funding for PHC does not match that of non-remote communities. For example, expenditure on General Practitioner (GP) services in very remote areas of Australia is just 57% of that in major cities [4]. At the same time, residents from remote areas of the NT are 50% more likely to be hospitalised than those from non-remote areas [5].

*Medicare*, the cornerstone of Australian PHC, is a fee-for-service funding model that reimburses general practitioners for services. In some cases, this funding is supplemented by performance incentive payments designed to encourage improved patient outcomes. Supported by relatively small contributions from state and federally funded community health, Indigenous health and mental health programs, *Medicare* underpins the majority of PHC in Australia. In remote areas, most services are provided not by GPs but by Remote Area Nurses (RAN) or Aboriginal Health Practitioners (AHP), resulting in less than a third of health clinic costs being claimable under Medicare [6]. The remuneration value placed on Medicare Benefits Schedule (MBS) items is derived from expenditures involved in running a typical Australian medical practice and does not reflect the safety and equity considerations associated with geographical remoteness. [4].

Current funding arrangements are inadequate, complex, administratively cumbersome and involve a large number of short term grants to many small organisations [7]. These funding arrangements are a product of history, lack transparency, and fail to account for variable populations, geographical classifications, differential health needs, and varied costs in different locations [8–10]. Zhao et al. (2006) demonstrated that the average cost of a face-to-face consultation with a medical officer in a remote context was approximately three times that of the corresponding MBS benefit fee [11]. Significant inequity characterises the funding of PHC services, particularly in the most remote areas of the country. To some degree, this disparity in expenditure is offset by Commonwealth funded grants to Aboriginal Controlled Community Health Organisations, which provide services to a large number of remote and very remote communities.

Responding to this inequity requires a transparent and systematic approach to funding that addresses population size, health need, geographical remoteness, and provides consumers with some understanding of the services they can reasonably expect to receive [8, 12]. Recommendations taking account of these factors have been made over many years, most notably by the National Health and Hospitals Reform Commission in 2009 [13, 14] and the OECD review of health care quality in Australia in 2015 [15]). Unfortunately these have gone largely unrealised.

Ensuring equitable health outcomes through more equitable access to PHC clearly requires equitable resourcing of these services so that they can respond appropriately to the needs of their catchment populations. This issue is complex, long-standing [16] and not unique to Australia. In developing countries there are few health services in rural and remote areas, and people experience a disproportionate burden of disease as a result [17]. The European Region is also faced with the challenge of providing quality health services at higher costs to the sparsely populated areas of Scandinavia. [18]. Our purpose here is not so much to review the extent of rural health inequities that characterise both developed and under-developed countries [19], but rather to consider how best to determine what level of PHC resourcing is required to benchmark core PHC services in communities of different sizes and composition in remote areas.

In an attempt to achieve this goal, important research has been undertaken to provide empirical evidence upon which policy-makers can develop more equitable funding models and workforce programs. Studies have defined which PHC services residents of rural and remote communities should be able to access [20], and have identified the population thresholds at which those services should be available from a resident (as opposed to visiting) service provider [21]. This study seeks to advance this research by investigating the resource levels required to deliver accessible, high quality PHC services in remote areas of the NT. In particular, this pilot study aimed to ascertain what funding is required to ensure equitable access to sustainable, high quality PHC irrespective of geographical isolation of the resident population in remote communities

## Methods

Data from the Australian Primary Care Collaboratives Program (APCC) were used to identify high performing remote PHC services. Five improvement measures describing the management of type two diabetes and coronary heart disease (two of the most salient indicators of PHC) were used to measure service performance. Improvement measures for diabetes included the proportion of registered patients with an HbA1c ≤ 7.0 mmol/l, with measured total cholesterol <4.0 mmol/l or LDL ≤ 2.0 and with last recorded blood pressure ≤ 130/80. Measures for coronary heart disease included the proportion of patients prescribed an anti-platelet agent and a statin. Practices were assigned an average trend rating by the APCC, which indicated whether a practice had improved, declined or stayed the same over a 12 month period. Based on their rating, health services were selected for communities that fell within three different-sized population groups (100–500, 501–1000 and 1001 to 3000). From a total of 743 practices that had collected data over an 18 month period, 31 remote or very remote services were eligible for inclusion. Of these, 16 were selected on the basis of meeting high quality performance criteria validated by expert opinion.

Services were then invited to participate in the study with 11 agreeing. After acceptance, a survey tool was forwarded to participants for completion. Data collected for the 2013–14 financial year included the types of PHC services provided, as defined by Thomas et al. (2015) [20], whether the services were provided by resident or visiting personnel [21], level of service utilisation, operating and capital expenditure and information describing the context of service delivery (see Table 1). Costs attributed to Public Health and capital and corporate projects were excluded from the data. All costs were adjusted for inflation to 2013–14 amounts [22]. A follow-up site visit and interview were conducted with each service to validate the survey data and ensure an accurate understanding of the health service environment.

Data identifying the number, duration, type and costs of visiting PHC services to remote communities were obtained from four visiting service providers, three territory wide services and one regional service. Data were combined across two broad categories; technical services (requiring significant technical infrastructure and maintenance) and non-technical services. An average cost per day for the two categories of visiting services was calculated and applied to the annual number of visit-days reported to obtain an estimated cost of visiting services.

Population data relevant to the catchment of each participating health service were obtained from the Australian Bureau of Statistics 2011 Census [23] including estimated resident population, age, Indigenous status, English spoken at home and workforce participation (Table 1).

Due to non-normality in many of the study variables, statistical analysis of associations has been via simple linear and quadratic regression with formal statistical inference (*p*-values) in the linear regression derived via the nonparametric bootstrap. Predictive ability of the resulting model was evaluated through the model R^2^ by testing against actual and proposed remote health funding levels reported in the “*Evaluation of the Child Health Check Initiative and the Expanding Health Service Delivery Initiative: Final report”* [24]*.*

**Table 1:** Summary of Remote Health Centre Characteristics

Expenditure	
• workforce or staff	• drugs, medical consumables and equipment
• professional development	• accounting / legal/ insurance expenses
• recruitment	• other
• communications	• corporate cost allocation
• vehicle operation	• capital and depreciation
• buildings	
ABS 2011 Population Demographics	
	• workforce Participation
• catchment population	• Socio-Economic Indexes for Areas (SEIFA)
• age	• travel time to nearest regional centre
• Indigenous status	• distance to nearest regional centre
• English spoken at home	• Australian Standard Geographical Classification – Remoteness Area (ASGC-RA)
Service Delivery	
• consultations per annum	• resident service provided
• patients currently registered	• visiting service provided
• patients seen in past 6 months	• regularity and duration of visiting services

Ethics approval was obtained from the Central Australian Human Research Ethics Committee (HREC-12-57). Data analysis was undertaken using IBM SPSS Statistics for Windows [25].

## Results

### Participant profile

The study included 11 remote NT health centres operated by four health service providers some of which provided outreach services to smaller communities or outstations. Catchment populations for these centres varied from 155 to 2124 and catchment areas extended to 64,000 km^2^. Some centres experienced considerable changes in population due to tourism in the ‘dry season’ or due to restricted mobility in the ‘wet season’.

Most residents in the service catchment populations were Indigenous, did not speak English at home and had low workforce participation. This demography changed slightly in those communities influenced by tourism and/or mining. Based on the Australian Standard Geographical Classification Remote Areas (ASGC-RA), all communities, including island settings, were classified as RA4 (remote) or RA 5 (very remote). The distance from communities to a public hospital varied considerably from 2 km to 560 km.

All centres provided a similar range of services, consistent with the scope of practice of RANs, AHPs and remote GPs. Four centres did not offer 24 h emergency care. In all cases where podiatry, physiotherapy, speech pathology, psychology, audiology and occupational therapy were available, they were provided by a visiting health professional. Visiting services data covered 2063 visits by 23 different health professions to 80 different remote communities over 4705 days.

All services reported costs particular to their remote locality. Some related specifically to their geographical location, while others were similar across several remote sites. Examples include: overtime costs of $220,000 per year associated with a 24 h service, accommodation for staff at $40,000 per year, satellite internet connection to support patient information systems of over $50,000 (in a small service with a population under 300), air travel costs for staff of $150,000 per annum and freight costs of $20,000 per annum.

**Table 2:** Summary of simple linear regression derived via the nonparametric bootstrap

Parameter	Intercept	Slope (SE)	*p*-value	Adjusted R^2^
Total annual cost of resident services	1,251,893.92	1698.83 (58)	0.004	0.72
Cost per capita	6268.303	−2.315 (−0.321)	0.015	0.47
Cost per consultation	211.58	−0.022 (0.008)	0.698	−0.075
Consultations per capita	29.59	−0.011 (0.002)	0.032	0.570
RANs per 1000 population	13.40	−0.006 (−0.001)	0.149	0.369
Visiting days per annum	61.78	0.253 (0.005)	0.021	0.728
Total annual cost of resident and visiting services	1,378,870.85	2600.00 (112)	0.001	0.882

### Resident services

**Fig. 1:**
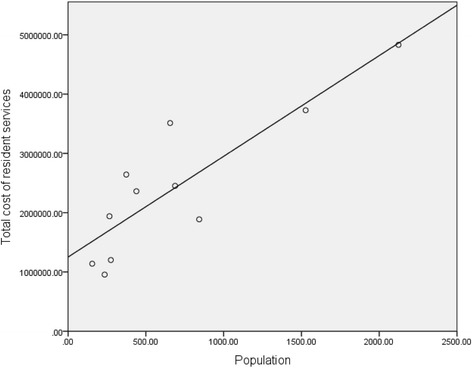
Cost of Resident Services by Population: linear regression using total cost of resident services for 11 primary health care services is used to predict cost of resident primary health care using population size. A significant relationship between cost and population is shown where the cost of resident services rises by $1698.83 per head of population per annum

Total costs of resident PHC services ranged from $955,603 to $4,830,823 per annum. There was a direct linear relationship with population size. A linear regression with bootstrapped statistical inference was calculated to predict the cost of resident services based on population size (Table 2, Fig. 1). A significant regression equation relationship was found. The cost of resident services is equal to $1,251,893.92 + ($1698.83 x population) per annum.

**Fig. 2:**
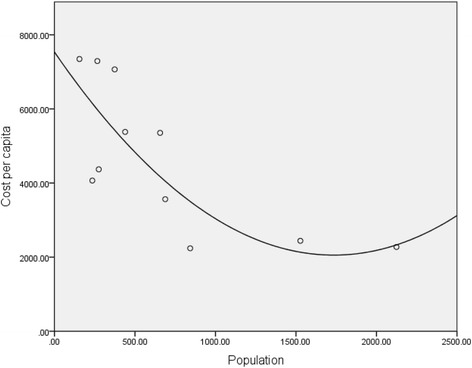
Per Capita Cost of Resident Primary Health Care Services in 11 Remote Communitites: per capita costs were determined using total cost of resident services and catchment population for each the 11 remote primary health care services. The data suggest the per capita costs decrease with increasing population in a non-linear relationship. This departure from linearity failed to reach statistical significance but may be supported by a larger sample

The cost of resident PHC services ranged from $2239 to $7346 per capita per annum, with larger services having significantly lower per capita costs (Table 2, Fig. 2). Figure 2 indicates non linearity where the reduction in cost per capita, with increasing population, levels off after a population of approximately 800 residents. A departure from a linear relationship is not clear in a quadratic regression with bootstrapped statistical inference (B = −6.318, *p* = 0.78), however would potentially become clearer with a larger sample.

**Fig. 3:**
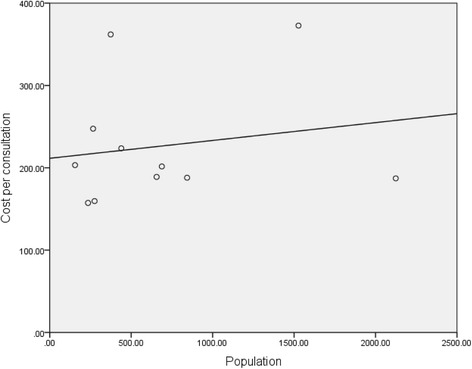
Mean Cost of One Resident Primary Health consultation in 11 Remote Communities: mean cost per consultation was determined using total cost of resident services and the number of consults provided per annum for each the 11 remote primary health care services. Linear regression shows no clear relationship between cost per consultation and population

The cost of resident PHC services per consultation showed considerably smaller range (between $157 and $372), and exhibited no significant association with population size (Table 2, Fig. 3).

**Fig. 4:**
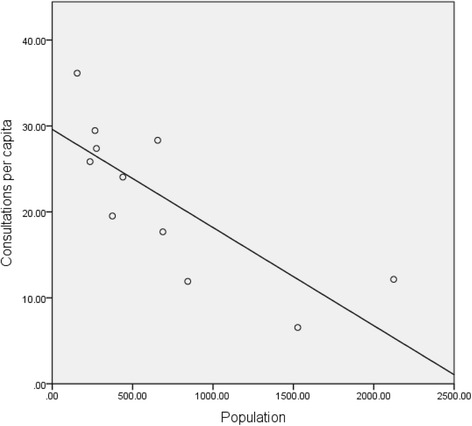
Mean Number of Consultations Per Capita in 11 Remote Communities: The mean number of consultations per capita was determined using the number of consultations per year and the catchment population for each remote primary health care service. Linear regression demonstrates a significant relationship between the number of consultations per capita per year and the catchment population for 11 primary health care services

Participating services provided between seven and 36 consults per capita per annum (Fig. 4). Smaller communities provided a significantly higher number of consultations per capita (Table 2).

The number of RANs per 1000 population showed an inverse relationship with population. While this relationship was not significant with a bootstrapped statistical inference (Table 2), statistical effect is indicated by the standardised coefficient (β = 0.657, *p* = 0.028) and a larger sample would be expected to show this relationship with greater certainty. Clinics in smaller communities had more resident nurses/clinical staff per 1000 population than larger communities. With the exception of one outlier, the number of nurses per 1000 population in participating services ranged from 2.8 to 12.7.

### Visiting services

**Fig. 5:**
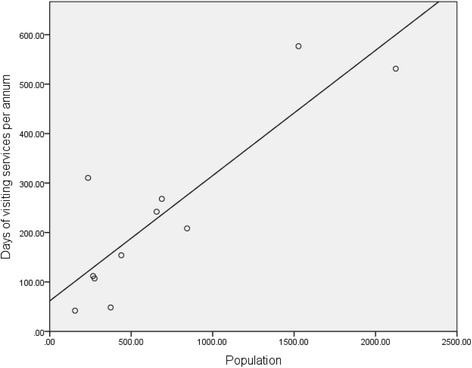
Days of Visiting Services per Annum in 11 Remote Communities: The self reported number of days of visiting services provided to each of 11 remote primary health care services are presented in a simple linear regression showing a significant relationship with population where the number of visiting days per annum increases with the catchment population of the health service

Visiting services included allied health, dental services, aged care assessment and case management services, alcohol and other drugs, and mental health and counselling services. The average total cost of delivering visiting PHC services was $4023.02 per day. The average cost per day was greater for services requiring significant infrastructure and maintenance (technical services) such as dental and audiology services ($6811.06) than for other allied health services ($2221.23). The mean duration of a visit was 2.28 days. The number of days of visiting services received per year increased significantly with population (Table 2, Fig. 5).

**Fig. 6:**
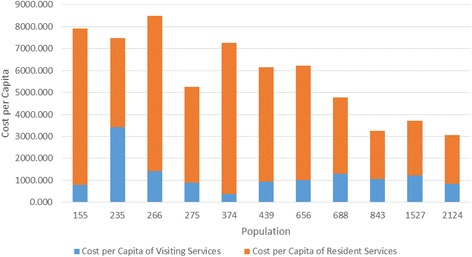
Cost Per Capita of Resident and Visiting Primary Health Care Services in 11 remote Communities: per capita costs of resident services are shown cumulatively with per capita cost of visiting services for each the 11 remote primary health care services. Total per capita costs decrease with increasing population

Visiting services provided the equivalent of between 0.5 and 1.6 full time staff per 1000 population, with the exception of one small community receiving the equivalent of five full time staff per 1000 population. The additional visiting services received by this community appear to have a compensatory effect to the relatively lower expenditure on resident services (see Fig. 6).

**Fig. 7:**
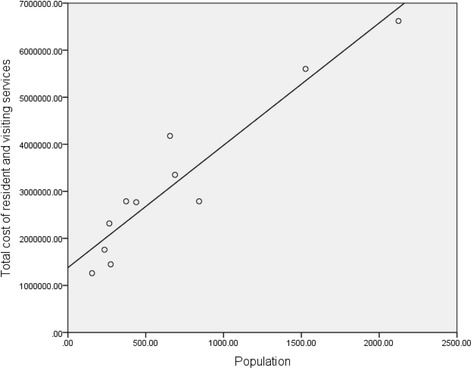
Total Cost of Resident and Visiting Services per Annum by Population: linear regression in which total cost of resident services for 11 primary health care services is used to predict cost of resident and visiting primary health care using population size. A linear relationship between cost of resident and visiting services and population is shown, where cost increases by $2600 per head per annum

A linear regression with bootstrapped statistical inference was calculated to predict cost of resident and visiting services based on population size (Table 2, Fig. 7). A significant regression equation was found with the cost of resident and visiting services being equal to $1,378,870.85 + ($2600.00 x population) per annum.

Cost estimates were compared with estimates provided by a number of other studies [26], and the actual expenditure reported by the Productivity Commission [27] (Table 3).

**Table 3:** Comparison with other estimates and actual expenditure

Study	Cost Per Consultation	Cost per Capita
Current study	227	4672
Ong et al. [26]	206	
Gador Whyte [33] Cost @ Min cost per CKD patient $4062 pa		4062
Zhao [11] Top Down Estimate per Consultation GP (inflation calculator applied to 2003/04 estimate)	220.55	
Zhao [11] Category 2 Single established disease, existing case (inflation calculator applied to 2003/04 $700)		918.97
AIHW [39] expenditure levels per episode of care for residents of remote and very remote areas	218	
Productivity Commission [27] - cost of community health services for Indigenous Australians during 2012/13 in the Northern Territory (inflation adjusted to 2013/14)		3647
Productivity Commission [27]- cost of community health services for Indigenous Australians real inflation adjusted average over 2008/09, 2010/11 and 2012/13 for the Northern Territory. (inflation adjusted to 2013/14)		6247
Evaluation of the Child Health Check Initiative and the Expanding Health Service Delivery Initiative [24] 2009–10 Average per capita EHSDI benchmark (across 10 remote sites) inflation adjusted to 2013/14		3822.67

Funding estimates, based on the predictive model developed in this study, were compared with data on health centre funding sourced from the “*Evaluation of the Child Health Check Initiative and the Expanding Health Service Delivery Initiative: Final report”* (EHSDI) [24] (Table 4).

**Table 4:** Comparison with EHSDI Actual and Benchmark Funding Levels

Population	Predicted income per Capita Proposed Model (2013/14)	EHSDI actual HSDA level funding (2009/10) - Inflation adjusted to 13/14	Difference	EHSDI HSDA level benchmark (inflation adjusted to 2013/2014)	Difference
2292	$2245.03	$2355.51	−4.92%	$3505.67	−56.15%
1527	$2518.67	$2706.52	−7.46%	$3283.81	−30.38%
2111	$2291.86	$3002.34	−31.00%	$3311.40	−44.49%
2124	$2288.23	$2730.80	−19.34%	$4017.83	−75.59%
688	$3518.44	$3559.76	−1.17%	$4358.91	−23.89%
1171	$2767.91	$2752.88	0.54%	$3578.52	−29.29%
926	$3050.77	$3440.54	−12.78%	$4431.76	−45.27%
348	$5296.23	$3077.39	41.89%	$3953.81	25.35%
235	$7026.04	$2928.38	58.32%	$3905.24	44.42%
Average	$3444.91	$2950.46	14.35%	$3816.33	−10.78%

## Discussion

Analysis of the relationships between expenditure, population size and nature of PHC activity suggest that there is a minimum funding base for a PHC service in remote communities in the NT, supplemented by a capitation rate. The analysis suggests that economies of scale take effect in communities with a catchment population of approximately 800.

Activity based funding involves payment on the basis of an expected number of episodes of care at a rate per episode [28]. In this study, activity-based costings (cost per consult) showed less variation than a capitation approach (the amount of health funds to be assigned per person for a service, over a given time, subject to national budget constraints. [29]), particularly when only cost of staff and medical supplies were included. As activity-based approaches foster a biomedical, episodic and piecemeal approach to patient care, focused solely on outputs rather than quality and outcomes, they are not appropriate as the sole funding criterion for remote contexts. Activity-based models may also threaten the viability of some PHC services due to their low volume of patients and the lack of flexibility required to meet local needs [15, 30].

Capitation cost is correlated with population size, offering some predictability. A significant correlation between the number of consults per capita and population might indicate that the higher cost of health services in smaller communities is associated with higher levels of service and fewer economies of scale, as well as reflecting variation in the pattern of need and epidemiology across remote communities. The finding that services with smaller catchment populations are providing significantly higher levels of service to their communities may be attributed to resourcing decisions for small health centres being determined more by safety and equity considerations than implied demand. That is, there is a minimum funding requirement to ensure safety of staff and residents in small isolated communities. Zhao et al. (2006) [11] report that the minimum feasible operation for a remote PHC centre included a half-time visiting medical officer, two RANs, one AHP and a minimum level of other supporting personnel. The cost of this is more than twice that of a typical practice on which the current MBS item costings are based.

This relationship between population (as a determinant of demand) and equity and safety considerations can be explained using the linear regression models in this study. Our modelling suggests a constant base level of funding per annum ($1,251,893.00 for resident services and $1,378,870.00 for resident and visiting services) that ensures quality and safety in addition to a per capita rate per annum which reflects community demand ($1698.83 for resident services and $2600.00 for resident and visiting services).

While the small number of large services in this study limit the conclusions that can be drawn from the observed levelling out of cost per capita in populations over 800, this observation was also noted by Zhao et al. (2010) [6] who identified that larger health centres, catering for populations greater than 700 start to see this efficiency gain.

Influenced by safety and equity considerations, efficiencies associated with scale decrease significantly for communities with populations under 200. Following the model derived in this study, the cost of resident PHC services increases by 79% as the population decreases from 200 ($7958 per capita) to 100 ($14,218 per capita). Estimates based on average staffing levels indicate that the minimum feasible service proposed by Zhao will meet the needs of populations up to 210–250. Cost effectiveness must be weighed against access and equity issues to determine the best models of care for such small populations. In many regions these communities are serviced by a minimum establishment of resident AHPs and/or an outreach service from larger neighbouring communities. In these cases the formula may be applied to the aggregate rather than individual community populations.

### Comparison of results with other studies

The average cost per consult identified in this study was $227 and the average cost of resident services per capita per annum was $4672. Costs were comparable (within 18%) with inflation adjusted estimates by Ong, Gador Whyte (minimum cost per CKD patient), Zhao (top down estimated cost per consultation), AIHW (expenditure levels per episode of care for residents of remote and very remote areas), and Allen and Clarke. Zhao’s estimated costs for per capita treatment of chronic disease were however significantly lower than the data in this study (Table 3).

Per capita cost of $4672 per annum was comparable with the Indigenous expenditure reports for the Northern Territory Community Health Services produced by the Productivity Commission [27, 31]. While the 2012/13 expenditure adjusted for inflation to 2013/14 was $3647 per capita (22% lower than the $4672 average identified in this study), the average inflation adjusted per capita expenditure across 2008/09, 2010/11 and 2012/13 was $6247 (34% higher than the average identified in this study), indicating a significant reduction in real terms of funding between 2008 and 2013.

Comparisons were made between estimated funding based on the predictive model developed in this study and data on health centre funding sourced from the “*Evaluation of the Child Health Check Initiative and the Expanding Health Service Delivery Initiative: Final report”* (EHSDI) [24]*.* Results indicated some consistency between the inflation-adjusted 2009/10 actual funding levels and those predicted by the 2013/14 model, although some services were identified as significantly under-resourced (Table 4).

The average funding under the model identified in this study varied by less than 15% from both the inflation adjusted actual 2009/10 and EHSDI benchmark funding level; although individual services vary significantly, particularly in relation to the EHSDI benchmark. EHSDI benchmarks were developed and applied to larger health service delivery areas. This benchmark was developed using national average MBS payment per capita and weightings for remoteness, English fluency and costs of service delivery in the NT. This approach does not address allocation of these resources between discreet services within the health service delivery area, nor economies of scale and so does not address the issues of minimum requirements for safety and equity identified in this study. As such there is greater disparity between EHSDI HSDA benchmarks as they are applied to individual services and those developed in this study, most noticeably in larger services where the effect of minimum requirements are dissipated by economies of scale.

The estimates derived from our model compare favourably with the majority of estimates found in other studies. These estimates were derived from a range of different methods, yet there is a broad consistency in the results.

### Limitations

Although a bottom-up approach is a more precise and reliable approach to costing health services [32], it is time consuming and expensive [33]. Ultimately decisions about selecting a costing method involve a trade-off between accuracy and resources available. This pilot study utilised a ‘top-down’ approach, with a small number of participants but incorporated a high level of detail and context to improve comparability between service costs.

While the small sample size limited the reliability and generalisability of our results, they have enabled researchers to further understand the complex financial context characterising remote health service delivery. Thorough interrogation of data was required to ensure it met the requirements of the research question, provided adequate consistency across services to validate comparisons and to explain any anomalies.

Fiscal equalisation requires adjustments in order to account for the various impacts of population size, geographical remoteness, diseconomies of scale, population density, Indigeneity, socio-economic status, health status and other variables which impact on both demand for, and cost of, health services [34–36]. Noting the relative homogeneity of services (predominantly Indigenous, grant funded and the sole service in a discrete catchment area) and the lack of consistent and reliable local level data, this study was unable to adequately evaluate the impact of all of these variables on resource requirements.

## Conclusions

Neither the Medicare system nor the current system of grants for funding remote PHC services provide an equitable approach to funding the health needs of Australians living in remote communities [6–9, 12, 37]. If we are to achieve equity in access to PHC for residents of remote areas, a transparent, planned and coordinated approach to funding is vital.

This study aimed to contribute to improved fiscal equalisation; an important response designed to address the problem of inequity in health care that spans all the dimensions of access [38]. The research findings contribute to an evidence base for funding remote PHC services that includes the safety and equity requirements for a minimum viable service. Although the sample size and unavailability of local level data provide some limitations, comparisons with other findings and approaches add some weight to the findings emanating from our model.

Any approach to resource the provision of PHC in remote communities will inevitably be constrained by the total resources available, not only how they are distributed. A consistent and rigorous approach to addressing clinical priorities, access and equity as they occur in small isolated communities located in remote environments will always be required. This study has demonstrated that a rigorous evidence-based and equitable approach to funding is possible and can underpin a transparent, reliable, planned and coordinated approach to the delivery of sustainable, high quality PHC in remote communities. More equitable access to PHC will contribute to reductions in the disproportionally high morbidity and mortality rates in these communities.

## Abbreviations

AHP: Aboriginal Health Practitioner
AIHW: Australian Institute of Health and Welfare
APCC: Australian Primary Care Collaboratives
ASGC-RA: Australian Standard Geographical Classification – Remoteness Area
CKD: Chronic Kidney Disease
EHSDI: Expanding Health Service Delivery Initiative
FTE: Full Time Equivalent
GP: General Practitioner
HSDA: Health Service Delivery Area
MBS: Medical Benefits Scheme
OECD: Organisation for Economic Cooperation and Development
PHC: Primary Health Care
RA: Remoteness Area
RAN: Remote Area Nurse
SEIFA: Socio-Economic Indexes for Areas
